# Supplementary material for: Comprehensive glycoproteomics shines new light on the complexity and extent of glycosylation in archaea
Source: PLoS Biol. 2021 Jun 17;19(6):e3001277. doi: 10.1371/journal.pbio.3001277 (PMC8241124; doi:10.1371/journal.pbio.3001277)
Supplement: S1 Fig — Samples from WT, Δagl15, and ΔaglB strains grown to mid-logarithmic and early-stationary growth phase (mixed using equal culture volumes) were fractionated into Mem, Cyt, and culture SN. Equal protein amounts for each strain (7.5 μg) were separated by LDS-PAGE and stained using Coomassie brilliant blue (A) or Pro-Q Emerald 300 glycoprotein staining (B). As controls, BSA (5 μg), which does not exhibit N-glycosylation but shows staining by periodic acid–Schiff staining [56], and the Rhodobacter capsulatus CcmG (15 μg) recombinantly expressed and purified from Escherichia coli, representing a non-glycosylated protein, were used. UV light exposure times have been adjusted to 0.1 seconds to result in minimal signal from the non-glycosylated control. The band corresponding to SLG has been marked (arrow), as its electrophoretic mobility is well established [51,52]. The images are representative for 2 biological replicates. Different staining procedures were performed on separate gels using the same biological replicates. The original images can be found in S1 Raw Images. Cyt, cytosol; Mem, membrane; SLG, S-layer glycoprotein; SN, supernatant; WT, wild-type. (PDF) [file pbio.3001277.s001.pdf]

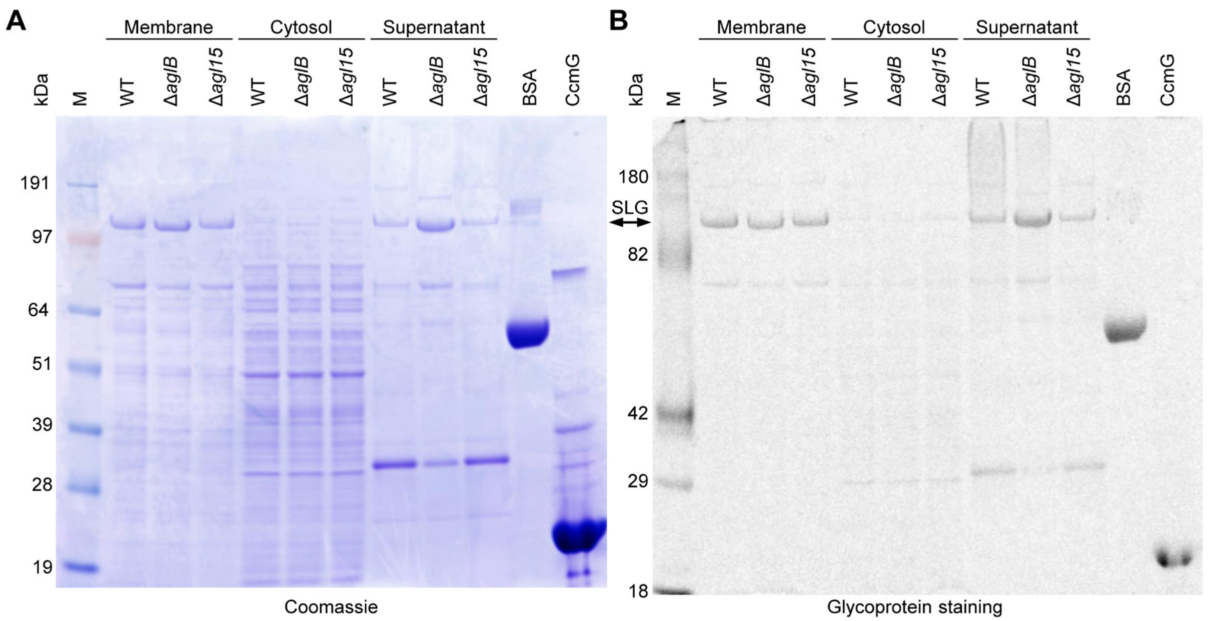

S1 Fig. Proteins from cellular fractions of WT, Δ*agl15* and Δ*aglB* exhibit comparable glycoprotein staining. Samples from WT, Δ*agl15* and Δ*aglB* strains grown to mid-logarithmic and early-stationary growth phase (mixed using equal culture volumes) were fractionated into membrane, cytosol and culture supernatant. Equal protein amounts for each strain (7.5 µg) were separated by LDS-PAGE and stained using Coomassie brilliant blue (**A**) or Pro-Q Emerald 300 glycoprotein staining (**B**). As controls, BSA (5 µg), which does not exhibit *N*-glycosylation but shows staining by periodic acid-Schiff staining [1], and the *Rhodobacter capsulatus* CcmG (15 µg) recombinantly expressed and purified from *Escherichia coli*, representing a non-glycosylated protein, were used. UV light exposure times have been adjusted to 0.1 s to result in minimal signal from the non-glycosylated control. The band corresponding to SLG has been marked (arrow), as its electrophoretic mobility is well established [2,3]. The images are representative for two biological replicates. Different staining procedures were performed on separate gels, using the same biological replicates. The original images can be found in S1 Raw Images.

References

1. Frenkel-Pinter M, Shmueli MD, Raz C, Yanku M, Zilberzwige S, Gazit E, et al. Interplay between protein glycosylation pathways in Alzheimer's disease. Science Advances. 2017;3: e1601576. doi:10.1126/sciadv.1601576
2. Abdul Halim MF, Karch KR, Zhou Y, Haft DH, Garcia BA, Pohlschroder M. Permuting the PGF Signature Motif Blocks both Archaeosortase-Dependent C-Terminal Cleavage and Prenyl Lipid Attachment for the Haloferax volcanii S-Layer Glycoprotein. Journal of Bacteriology. 2016;198: 808–815. doi:10.1128/JB.00849-15
3. Abdul-Halim MF, Schulze S, DiLucido A, Pfeiffer F, Filho AWB, Pohlschroder M. Lipid Anchoring of Archaeosortase Substrates and Midcell Growth in Haloarchaea. mBio. 2020;11. doi:10.1128/mBio.00349-20
