# Supplementary material for: Comprehensive glycoproteomics shines new light on the complexity and extent of glycosylation in archaea
Source: PLoS Biol. 2021 Jun 17;19(6):e3001277. doi: 10.1371/journal.pbio.3001277 (PMC8241124; doi:10.1371/journal.pbio.3001277)
Supplement: S2 Fig — Annotated spectra for SLG N-glycopeptides comprising the peptide sequence VGIANSSATNTSGSSTGPTVE with AglB- (A) and Agl15-dependent (B) N-glycans attached to the N-glycosites N274 and N279. Measured raw peaks are shown in gray, annotated a- and b-ions in purple, y-ions in yellow, and N-glycopeptide-specific Y- and B-ions in cyan. Insets illustrate the peptide sequence coverage through a- or b-ions (purple) and y-ions (yellow) (in both cases detected ions shown as wide bar, missing ions shown as line), as well as the coverage of Y- and B-ions (detected ions shown in cyan). The underlying source data for A and B can be found in S1 Data. MS2, tandem mass spectrum; SLG, S-layer glycoprotein. (PDF) [file pbio.3001277.s002.pdf]

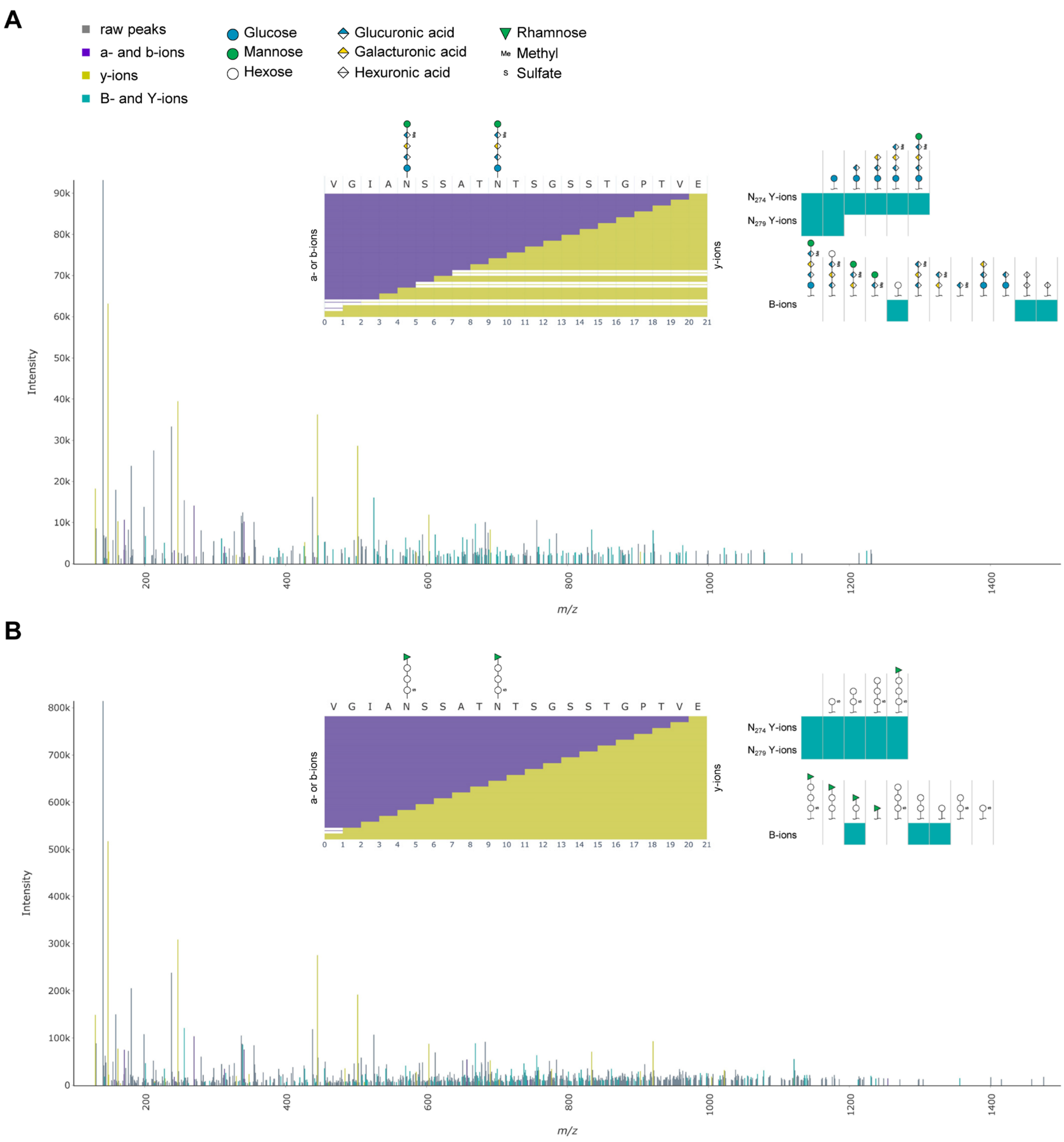

S2 Fig. MS2 spectra of SLG *N*-glycosites N274 and N279 strongly support modification by AglB- as well as Agl15-dependent *N*-glycans. Annotated spectra for SLG *N*-glycopeptides comprising the peptide sequence VGIANSSATNTSGSSTGPTVE with AglB- (**A**) and Agl15-dependent (**B**) *N*-glycans attached to the *N*-glycosites N274 and N279. Measured raw peaks are shown in grey, annotated a- and b-ions in purple, y-ions in yellow and *N*-glycopeptide-specific Y- and B-ions in cyan. Insets illustrate the peptide sequence coverage through a- or b-ions (purple) and y-ions (yellow) (in both cases detected ions shown as wide bar, missing ions shown as line), as well as the coverage of Y- and B-ions (detected ions shown in cyan). The underlying source data for A and B can be found in S1 Data.
