# Supplementary material for: Comprehensive glycoproteomics shines new light on the complexity and extent of glycosylation in archaea
Source: PLoS Biol. 2021 Jun 17;19(6):e3001277. doi: 10.1371/journal.pbio.3001277 (PMC8241124; doi:10.1371/journal.pbio.3001277)
Supplement: S3 Fig — The number of identified peptides (light gray) and proteins (dark gray) for each dataset is shown as a barplot (sorted by the total number of identified proteins). The underlying source data can be found in S1 Data. ArcPP, Archaeal Proteome Project. (PDF) [file pbio.3001277.s003.pdf]

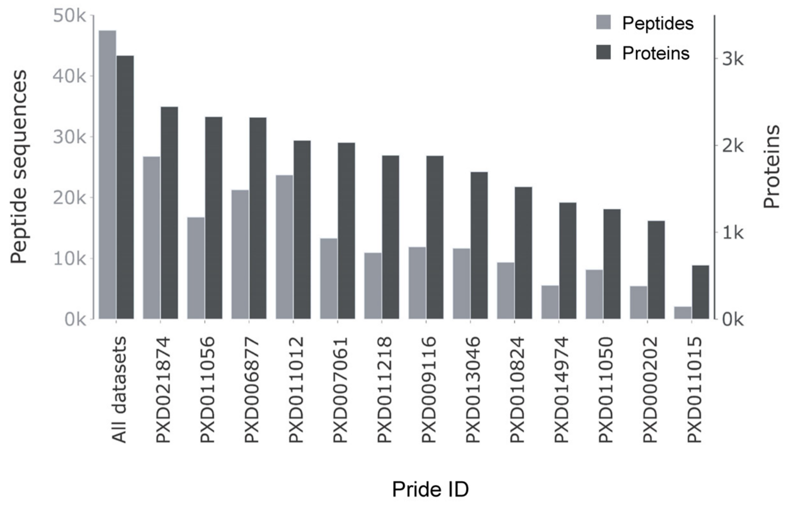

S3 Fig. More proteins and peptides have been identified in PXD021874 than in any other dataset of the ArcPP. The number of identified peptides (light grey) and proteins (dark grey) for each dataset is shown as a barplot (sorted by the total number of identified proteins). The underlying source data can be found in S1 Data.
