# Supplementary material for: Comprehensive glycoproteomics shines new light on the complexity and extent of glycosylation in archaea
Source: PLoS Biol. 2021 Jun 17;19(6):e3001277. doi: 10.1371/journal.pbio.3001277 (PMC8241124; doi:10.1371/journal.pbio.3001277)
Supplement: S4 Fig — Shown is an annotated spectrum for the Ths3 N-glycopeptide with sequence KSEVDTEYNITSVDQLTAAIDAEDSELR harboring an Agl15-dependent N-glycan. Measured raw peaks are shown in gray, annotated a- and b-ions in purple, y-ions in yellow, and N-glycopeptide-specific Y- and B-ions in cyan. Insets illustrate the peptide sequence coverage through a- or b-ions (purple) and y-ions (yellow) (in both cases detected ions shown as wide bar, missing ions shown as line), as well as the coverage of Y- and B-ions (detected ions shown in cyan). The underlying source data can be found in S1 Data. MS2, tandem mass spectrum; Ths3, thermosome subunit 3. (PDF) [file pbio.3001277.s004.pdf]

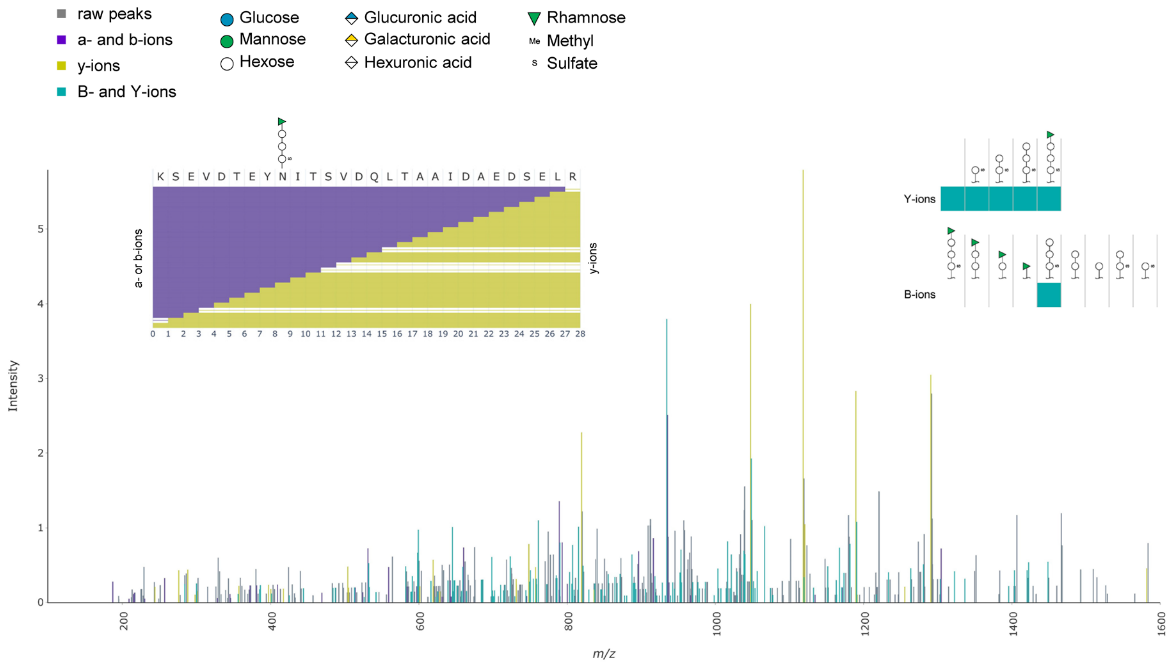

S4 Fig. AgI15-dependent *N*-glycosylation of Ths3 is strongly supported by MS2 fragment ion series. Shown is an annotated spectrum for the Ths3 *N*-glycopeptide with sequence KSEVDTEYNITSVDQLTAAIDAEDSELR harboring an AgI15-dependent *N*-glycan. Measured raw peaks are shown in grey, annotated a- and b-ions in purple, y-ions in yellow and *N*-glycopeptide-specific Y- and B-ions in cyan. Insets illustrate the peptide sequence coverage through a- or b-ions (purple) and y-ions (yellow) (in both cases detected ions shown as wide bar, missing ions shown as line), as well as the coverage of Y- and B-ions (detected ions shown in cyan). The underlying source data can be found in S1 Data.
