# Supplementary figures and images for: Comprehensive glycoproteomics shines new light on the complexity and extent of glycosylation in archaea
Source: PLoS Biol. 2021 Jun 17;19(6):e3001277. doi: 10.1371/journal.pbio.3001277 (PMC8241124; doi:10.1371/journal.pbio.3001277)

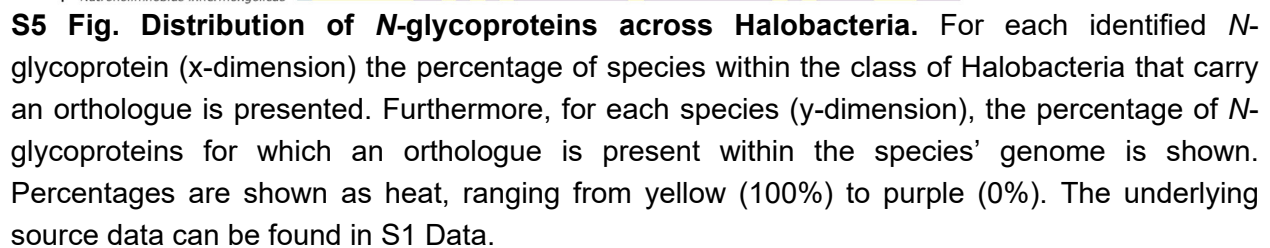

Supplement: S5 Fig — For each identified N-glycoprotein (x-dimension), the percentage of species within the class of Halobacteria that carry an orthologue is presented. Furthermore, for each species (y-dimension), the percentage of N-glycoproteins for which an orthologue is present within the species’ genome is shown. Percentages are shown as heat, ranging from yellow (100%) to purple (0%). The underlying source data can be found in S1 Data. (PDF) [file pbio.3001277.s005.pdf]

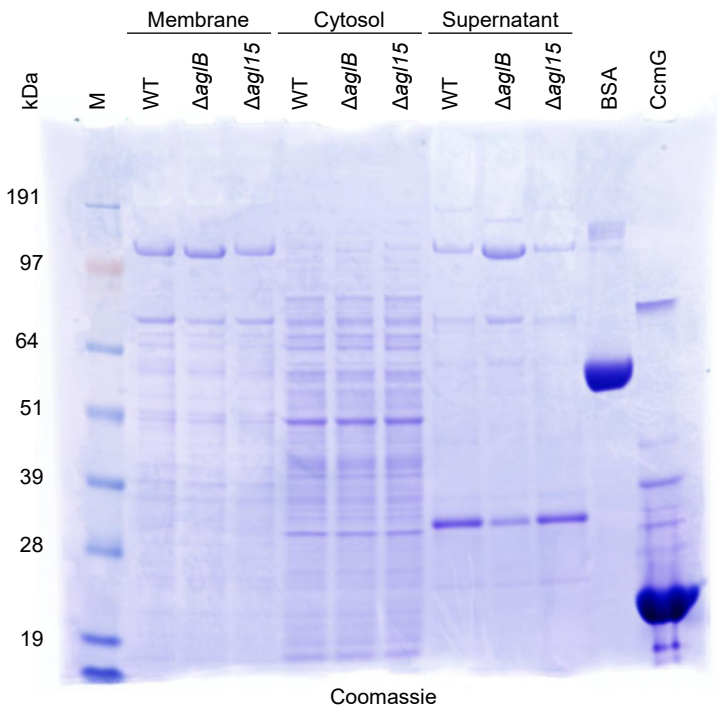

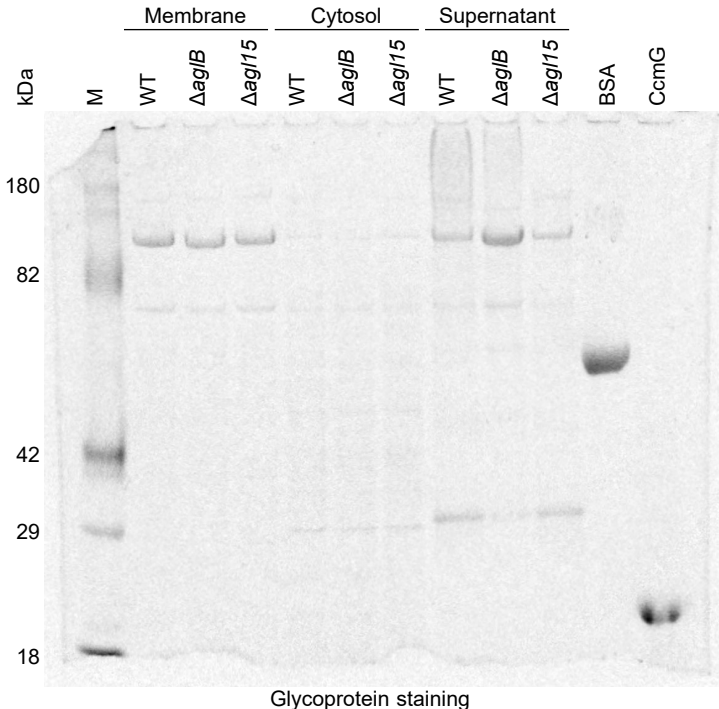

Supplement: S1 Raw images — Samples from WT, Δagl15, and ΔaglB strains grown to mid-logarithmic and early-stationary growth phase (mixed using equal culture volumes) were fractionated into Mem, Cyt and culture SN. Equal protein amounts for each strain (7.5 μg) were separated by LDS-PAGE and stained using Coomassie brilliant blue (page 1) or Pro-Q Emerald 300 glycoprotein staining (page 2). As controls, BSA (5 μg), which does not exhibit N-glycosylation but shows staining by periodic acid–Schiff staining [56], and the Rhodobacter capsulatus CcmG (15 μg) recombinantly expressed and purified from Escherichia coli, representing a non-glycosylated protein, were used. UV light exposure times have been adjusted to 0.1 seconds to result in minimal signal from the non-glycosylated control. Cyt, cytosol; Mem, membrane; SN, supernatant; WT, wild-type. (PDF) [file pbio.3001277.s008.pdf]
