# Supplementary material for: Comprehensive glycoproteomics shines new light on the complexity and extent of glycosylation in archaea
Source: PLoS Biol. 2021 Jun 17;19(6):e3001277. doi: 10.1371/journal.pbio.3001277 (PMC8241124; doi:10.1371/journal.pbio.3001277)
Supplement: S6 Fig — Annotated spectra for noncanonical N-glycosites within the peptide sequences VVWTSESGSNSATLQR (A) and MPSNANIMGVTPGSR (B) corresponding to the protein pilA6 and HVO_2160, respectively, are shown. Both N-glycopeptides are modified by an AglB-dependent glycan, and the site of attachment is indicated. Measured raw peaks are shown in gray, annotated a- and b-ions in purple, y-ions in yellow, and N-glycopeptide-specific Y- and B-ions in cyan. Insets illustrate the peptide sequence coverage through a- or b-ions (purple) and y-ions (yellow) (in both cases detected ions shown as wide bar, missing ions shown as line), as well as the coverage of Y- and B-ions (detected ions shown in cyan). The underlying source data for A and B can be found in S1 Data. MS2, tandem mass spectrum. (PDF) [file pbio.3001277.s006.pdf]

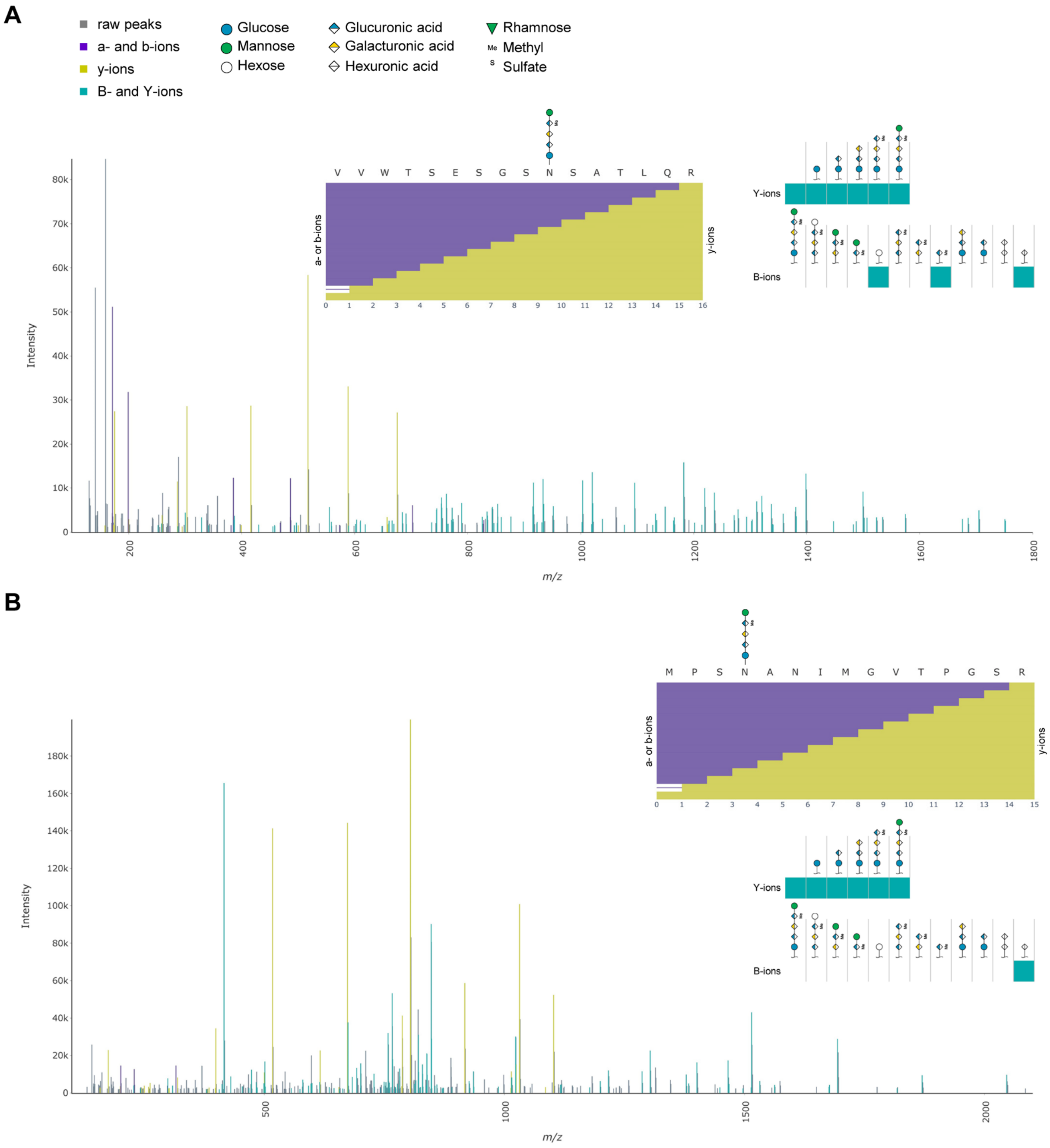

S6 Fig. *N*-glycopeptides with non-canonical *N*-glycosites are strongly supported by MS2 fragment ion series. Annotated spectra for non-canonical *N*-glycosites within the peptide sequences VVWTSESGS**N**SATLQR (**A**) and MPS**N**ANIMGVTPGSR (**B**) corresponding to the protein pilA6 and HVO_2160, respectively, are shown. Both *N*-glycopeptides are modified by an AglB-dependent glycan and the site of attachment is indicated. Measured raw peaks are shown in grey, annotated a- and b-ions in purple, y-ions in yellow and *N*-glycopeptide-specific Y- and B-ions in cyan. Insets illustrate the peptide sequence coverage through a- or b-ions (purple) and y-ions (yellow) (in both cases detected ions shown as wide bar, missing ions shown as line), as well as the coverage of Y- and B-ions (detected ions shown in cyan). The underlying source data for A and B can be found in S1 Data.
