# Supplementary material for: Comprehensive glycoproteomics shines new light on the complexity and extent of glycosylation in archaea
Source: PLoS Biol. 2021 Jun 17;19(6):e3001277. doi: 10.1371/journal.pbio.3001277 (PMC8241124; doi:10.1371/journal.pbio.3001277)
Supplement: S1 Table — For each protein that was identified to be N-glycosylated, the HVO ID, description, InterPro domains and arCOGlet classification are given together with results of phylogenetic and gene synteny analysis for the genes encoding the identified N-glycoproteins. (PDF) [file pbio.3001277.s007.pdf]

S1 Table. Analysis of phylogeny, gene synteny and protein domains of identified *N*-glycoproteins. For each protein that was identified to be *N*-glycosylated, the HVO ID, description, InterPro domains and arCOGlet classification is given together with results of phylogenetic and gene synteny analysis for the genes encoding the identified *N*-glycoproteins.

HVO ID	Description[1]	InterPro Domains[2]	arCOG let[3]	Taxonomic range of orthologs[4]	Halobacteria with orthologs[5]	Neighboring genes/ SynTax analysis[2]	Comments
**HVO_0307**	conserved hypothetical protein	none	N	Hal+	157	conserved gene clustering:<br><u>downstream:</u><br>HVO_0308 DUF1628 domain, pilin-related<br>HVO_0309 menG, menaquinone biosynthesis<br><u>upstream:</u><br>HVO_0306 probable transmembrane glycoprotein with HTH domain<br>HVO_0305/0304 etfA/B, subunits of an electron transfer flavoprotein	-
**HVO_0504**	DUF192 family protein	DUF192	S	Arc	152	genomic vicinity of HVO_0505 to HVO_0511 in several genomes but no strict gene synteny:<br><u>downstream:</u><br>HVO_0505 small, uncharacterized<br>HVO_0506 ABC transporter permease/ATPase, unassigned substrate<br>HVO_0507 putative amidohydrolase, creatininase domain<br>HVO_0508 uncharacterized<br>HVO_0509 NUDIX family hydrolase<br>HVO_0510 mptD, folate biosynthesis<br>HVO_0511 azf, glucose-6-phosphate 1-dehydrogenase	tempting to speculate that HVO_0504 is a substrate-binding protein for HVO_0506 or that HVO_0506 transports the product of a potential enzymatic reaction catalyzed by HVO_0504
**HVO_0778**	(Ths3) thermosome subunit 3	-	O	Arc	122	-	-
**HVO_0892**	(NosD) ABC-type transport system periplasmic substrate-binding protein (probable substrate copper)	-	P	Hal+	102	-	substrate assignment only tentative
**HVO_0972**	(PilA1) pilin PilA	DUF1628	N	Eur	107	gene neighborhood not conserved	-

**HVO_1014**	(CoxB1) cox-type terminal oxidase subunit II	-	C	Arc	136	-	-
**HVO_1030**	DUF4382 domain protein	DUF4382	S	Hal	32	gene neighborhood not conserved	-
**HVO_1176**	conserved hypothetical protein	none	S	Hal	157	part of a strictly conserved three-gene operon:<br><u>downstream:</u><br>HVO_1177 ADP-ribose pyrophosphatase<br><u>upstream:</u><br>HVO_1175 DUF2110 domain protein	-
**<u>Cluster I</u>**							
**HVO_1210**	(ArlA1) archaellin A1	-	N	Eur	131	-	-
**HVO_1211**	(ArlA2) archaellin A2	-	N	Eur	131	-	-
**HVO_1259**	conserved hypothetical protein	none	S	Hal	32	conserved gene clustering, 6-gene operon:<br><u>downstream:</u><br>HVO_1258 uncharacterized<br>HVO_1257 AAA-type ATPase, MoxR type<br>HVO_1256 vWFA domain<br><u>upstream:</u><br>HVO_1260 vWFA domain<br>HVO_1261 vWFA domain	-
**HVO_1530**	(AglB) dolichyl-monophosphooligosaccharide--protein glycotransferase AglB	-	M	Eur	157	-	-
**HVO_1624**	conserved hypothetical protein	none	S	Hal	33	one clustered gene:<br><u>upstream:</u><br>HVO_1622 ArsR family transcription regulator	-
**HVO_1673**	conserved hypothetical protein	none	S	Hal	148	gene neighborhood not conserved	-
**HVO_1749**	conserved hypothetical protein	none	S	Hal	98	gene neighborhood not conserved	-
**<u>Cluster II</u>**							
**HVO_1802**	peptidase M10 family protein	metalPep	E	Hal+	57	-	the more specific assignment to metallopeptidase M10 is found for paralog HVO_1580

**HVO_1806**	conserved hypothetical protein	none	S	Eur	156	gene neighborhood not conserved	-
**HVO_1870**	peptidase M50 family protein	pepM50	O;R	Arc	156	three of five downstream genes are genomically clustered:<br>HVO_1871 hemQ coproheme decarboxylase<br>HVO_1874 oxidoreductase domain<br>HVO_1875 O-acetyltransferase domain	-
**<u>Cluster III</u>**							
**HVO_1944**	probable transmembrane glycoprotein / HTH domain protein	none	K	Hal+	124	gene pairing conserved:<br>HVO_1944<br>HVO_1945 conserved hypothetical protein<br>no additional clustering	a HTH domain is assigned to close homolog C453_17564
**HVO_1945**	conserved hypothetical protein	none	N	Hal	92	see HVO_1944	-
**HVO_1976**	(SecD) protein-export membrane protein SecD	-	U	Eur	156	conserved gene pair:<br>HVO_1976 secD<br>HVO_1975 secF	-
**HVO_1988**	GATase domain protein	GATase	S	Hal	68	conserved gene pair:<br>HVO_1988<br>HVO_1987 sppA2 signal peptide peptidase<br>no additional clustering	-
**<u>Cluster IV</u>**							
**HVO_2062**	(PilA2) pilin PilA	DUF1628	N	Eur	107	gene neighborhood not conserved	genes for the Agl15-dependent *N*-glycosylation pathway are encoded directly downstream of pilA2 (HVO_2046 to HVO_2061)
**HVO_2066**	conserved hypothetical protein	none	M	Hal+	95	gene neighborhood not conserved	-
**HVO_2070**	conserved hypothetical protein	none	N	n/a	n/a	conserved gene pair:<br>HVO_2070<br>HVO_2069 RND family permease<br>no additional clustering	40% protein sequence identity to HVO_A0466
**HVO_2071**	probable secreted glycoprotein	none	M	Hal	3	clustering not analyzed (too rare)	-
**HVO_2072**	(Csg) S-layer glycoprotein	-	M	Hal	36	gene neighborhood not conserved	SLGs are highly diverse between species from the genus *Haloferax*; the SLG

							from *Hfx. gibbonsii* (24% protein sequence identity) has more orthologs (129)
**HVO_2074**	probable secreted glycoprotein	none	S	Hal	10	clustering not analyzed (too rare)	-
**HVO_2076**	probable secreted glycoprotein (nonfunctional)	DUF304	M	Hal	7	clustering not analyzed (too rare)	targeted by transposon; most likely, a truncated version of this protein is stable enough to permit its proteomic detection; orthoDB analysis based on a close, nondisrupted homolog; DUF304 assigned to close homolog D320_03753
**HVO_2081**	pectin lyase domain protein	pectin lyase	G;P	Hal	19	clustering not analyzed (too rare)	-
**HVO_2082**	conserved hypothetical protein	none	M	Hal+	95	part of a three-gene operon:<br>HVO_2083 ABC transporter ATPase<br>HVO_2084 ABC transporter permease	tempting to speculate that HVO_2083/2084 transports the product of a potential enzymatic reaction catalyzed by HVO_2082; alternatively HVO_2082 could be a substrate-binding protein for HVO_2083/2084; this is considered less likely (lower number of orthologs; several genomes code for homologs of permease/ATPase but not for a homolog of HVO_2082)
**HVO_2084**	ABC-type transport system permease protein (probable substrate macrolides)	-	V	Arc	157	see HVO_2082	-
<u>**Cluster V**</u>							
**HVO_2160**	probable secreted glycoprotein	Ig-like	M;O; S	Eur	9	clustering not analyzed (too rare)	-
**HVO_2161**	probable secreted glycoprotein	none	T	Hal	8	clustering not analyzed (too rare)	-

**HVO_2167**	DUF4350 domain protein	DUF4350 GATase	S	Arc	84	conserved gene clustering (11 genes):<br>downstream:<br>HVO_2166 conserved hypothetical protein<br>HVO_2165 ABC transporter permease<br>HVO_2164 ABC transporter permease<br>HVO_2163 ABC transporter ATPase<br>upstream (total six genes):<br>HVO_2168 AAA-type ATPase, MoxR type<br>HVO_2169 conserved hypothetical protein<br>HVO_2170 conserved hypothetical protein<br>HVO_2171 DUF58 domain protein<br>HVO_2172 conserved hypothetical protein<br>HVO_2173 DUF1616 domain protein	-
**HVO_2172**	conserved hypothetical protein	none	L;M	Hal+	84	see HVO_2167	-
**HVO_2173**	DUF1616 family protein	DUF1616	S	Eur	133	see HVO_2167	-
**Cluster VI**							
**HVO_2533**	conserved hypothetical protein	none	S	Eur	96	gene neighborhood not conserved	-
**HVO_2535**	conserved hypothetical protein	none	S	Hal	11	clustering not analyzed (too rare)	-
**HVO_2634**	conserved hypothetical protein	none	S	Hal	27	gene neighborhood not conserved	-
**HVO_A0039**	conserved hypothetical protein	none	-	Hal	5	-	-
**HVO_A0466**	conserved hypothetical protein	none	N	Eur	128	conserved gene pair:<br>HVO_A0466<br>HVO_A0467 RND family permease<br>no additional clustering	40% protein sequence identity to HVO_2070; SyntTax analysis performed with close homolog G3A49_05450
**HVO_A0499**	conserved hypothetical protein	none	-	Hal	12	-	-
**HVO_B0194**	LppX domain protein	LppX	M	Eur	86	gene neighborhood not conserved	SyntTax analysis performed with close homolog G3A49_07715
**HVO_C0054**	hypothetical protein	none	-	n/a	0	-	-

[1]the description term “conserved hypothetical protein” is used for proteins devoid of an InterPro domain assignment and for which functionally characterized homologs are absent or too distant for annotation transfer.
[2]with few exceptions, InterPro domains, neighboring genes and SyntTax analyses are not reported if the *N*-glycoprotein itself is well characterized. For plasmid-encoded proteins (codes start with HVO_A, HVO_B, or HVO_C), SyntTax analysis is not available, unless a close homolog from a related species is chromosomally encoded. Domain abbreviations: DUF, domain of unknown function; GATase, glutamine aminotransferase; metalPep, metallopeptidase; pepM50, MEROPS peptidase family M50; vWFA, von Willebrand factor type A.
[3]arCOGlet: C, energy production and conversion; E, amino acid transport and metabolism; G, carbohydrate transport and metabolism; K, transcription; L, replication, recombination and repair; M, cell wall/membrane/envelope biogenesis; N, cell motility; O, post-translational modification, protein turnover, chaperones; P, inorganic ion transport and metabolism; R, general function prediction only; S, function unknown; T, signal transduction mechanisms; U, intracellular trafficking, secretion, and vesicular transport; V, defense mechanisms
[4]taxonomic range: Hal, Halobacteria; Eur, Euryarchaeota; Arc, Archaea; Hal+ indicates few additional orthologs beyond Halobacteria.
[5]number of genomes from the taxonomic order Halobacteria which contain an ortholog; a total of 161 genomes are considered. The term “n/a” refers to proteins which are not assigned to a protein group in OrthoDB.
