# Supplementary material for: Comprehensive glycoproteomics shines new light on the complexity and extent of glycosylation in archaea
Source: PLoS Biol. 2021 Jun 17;19(6):e3001277. doi: 10.1371/journal.pbio.3001277 (PMC8241124; doi:10.1371/journal.pbio.3001277)
Supplement: Attachment — Submitted filename: 2021-04-20_glycoproteomics_response_to_reviewers.pdf [file pbio.3001277.s010.pdf]

We would like to thank both reviewers for their supportive and constructive comments and have provided a detailed response to each comment below.

Reviewer #1: General comments

The manuscript of Schulze et al. represent a long-overdue comprehensive analysis of the glycoproteome of the model archaeon Haloferax volcanii. It reveals interesting overlaps in glycosylation between the major (AglB-dependent) and the minor (Agk15-dependent) N-glycosylation pathways, and also hints at O-glycosylations. When combined with previous ArcPP data from these authors, there are 45 high-confidence glycoproteins, many of which are of unknown function. Overall, the methodology used is conservative, and therefore highly unlikely to erroneously identify a non-existing glycosylation, but the authors should acknowledge that for low abundance proteins glycosylation could be missed due to the stringency and those 45 probably represent a lower bound, especially since some membrane-associated proteins may be only highly expressed under specific conditions. The phenotypic characterization is convincing, but does not add much to what is already known about glycosylation mutants.

Specific comments

Thermosome subunit 3 is the only exclusively Agl15-dependent glycoprotein, the only cytosolic glycoprotein and is also o-glycosylated! The authors speculate that some of it may be surface associated (maybe as a moonlighting role that is not thermosome related) but I could not find indication as to whether the glycosylated peptides were exclusively primarily in the membrane and secreted fractions. I think that these 3 observations are unlikely to be coincidental. My null hypothesis (which may be wrong, but should at least be considered) is that being part of a chaperone complex implies that this could just be a protein that gets glycosylated at random because it is highly abundant and interacts closely with many proteins, including, presumably, glycosyl-transferases. I understand that the data from all fractions were pooled, but once high-confidence glycosylated peptides were detected one can go back to the individual protein-associated peptides and glycopeptides and see how many peptides are identified in the cytosolic vs. the membrane fractions. This is by no means truly quantitative, but should give some idea.

> **Unfortunately, *N*-glycosylated thermosome subunit 3 peptides were only identified in the dataset PXD011056, which analyzed whole cell extracts without subcellular fractionation. Therefore, we are unable to analyze whether the *N*-glycosylated peptides were exclusively or primarily found in the membrane or secreted fraction. Nevertheless, we checked for the number of non-glycosylated peptides and PSMs in datasets that analyzed subcellular fractions and found, across multiple datasets, that approximately the same number of non-glycosylated peptides and PSMs corresponding to Ths3 were identified in membrane samples as in cytosol samples. This further supports a dual localization and membrane association of Ths3. However, we now also noted that *N*-glycosylation of Ths3**

could be coincidental due to the interactions of thermosomes with other proteins. Given that Agl15-dependent *N*-glycosylation is postulated to occur on the exterior side of the plasma membrane, it should be noted though, that secretion of Ths3 would still be required for these interactions.

The phylogenetic analysis is appropriate and well-thought out. Nevertheless, it would have been interesting and informative to also show the fraction of Haloferax species/strains that encode those proteins.

The analysis of Haloferax species (as well as other Halobacteria) for orthologs of identified glycoproteins is shown as part of Supplementary figure S4.

Reviewer #2: Schulze et al. describe the glycoproteome of Haloferax volcanii, the most studied archaea to date. The authors made a significant contribution implementing mass spectrometry to identify peptides and proteins targeted by 2 known glycosylases from cell extracts. Among the main discoveries, authors elucidated the required presence of AglB for global N-glycosylation activity in the cell, and also the presence of Agl15-dependent N-glycosylation of proteins across different growth phases. The latter evidence suggests that Agl15 is not only important for salt-stress response but also steady-state maintenance of post-translational modifications. This method represents a new and powerful tool for future cell biology studies to map important pathways responsible for morphogenesis in haloarchaea.

Overall, the manuscript is well written and conclusions are clear and justifiable by the presented data. However, there are important controls and clarifications that are missing in the paper, especially regarding Figure 2.

Specific comments, as they show in the manuscript:

Line 86: Correct "archaealla"

This has been corrected to “archaella”

Lines 117-118: I recommend avoid overinterpretation from the observation that the agl15 mutant grows at higher ODs, provided readers could misunderstand that this reflects a significantly higher cell density. Because the difference is under 10%, other factors could be contributing to absorbance at higher ODs (e.g. scattering caused by secretion of extracellular matrix). Since this is not really relevant to the core claims of the paper, it would be safer to cut this segment from the manuscript or explain the implications in the discussion section (lines 522-523).

We have rephrased the corresponding sections in the results and discussion to avoid an overinterpretation of the results.

Lines 124-129: Differences in cell morphology look rather subtle. To really state these images are representative, authors should show any form of measurements to differentiate between rods and disks. I see that the corresponding author has successfully done this in a recent work (Abdul-Halim 2020). I also think it's remarkable that the lack of any N-glycosylation barely affected the aglB shape profile. Authors should mention the fact and put in context with past papers that show S-layer defects in different salt concentrations.

We have now included a quantitative analysis of cell shapes across the different conditions and strains, including a statistical analysis that confirmed differences in cell shape between the WT and *N*-glycosylation pathway mutants at different growth stages.
Furthermore, we have extended the discussion in regard to the effect of N-glycosylation on cell shape.

Lines 172-174: I am having trouble understanding some points of the following segment:

A) How did the authors identify which protein is the SLG? Is it the ~100kDa band? Please clarify and update the panel indicating SLG

The band corresponding to SLG has now been marked in the figure. The electrophoretic mobility of SLG has been well-established from previous publications.

B) Even though the authors calibrated the loading of the gel, it's not clear if coomassie is the best way to semi-quantitatively compare different fractions as the dye labels differently oxidated proteins and glyco groups, which can be on the way to detect shedding itself. In order to avoid these artifacts, authors should stain gels with a quantitative staining protocol, e.g. silver or fluorescent probes.

Since the results shown in regard to a reduced S-layer stability are only confirming results of previous publications, we have omitted a quantitative analysis. However, in order to reflect that the performed LDS-PAGE is only a pre-experiment for the performed in-depth glycoproteomic analysis, we have moved this figure into the supplemental materials.

C) Another problem with the above claim is the also observed enriched band of SLG in the aglB mutant. Using adequate staining will allow authors to get membrane:supernatant ratios to make the point there is (or not) enrichment in a specific fraction. The same measurement should be done in the glycoprotein staining.

Please refer to the response on comment B).

D) Additionally, authors should clarify if different staining in 2A and 2B were performed in the same or different gels. It's my understanding that it should be done in different gels, from both biological and technical triplicates to avoid staining artifacts.

> **Different staining procedures were performed on separate gels, run simultaneously in the same apparatus, using the same biological replicates. We have now added this information to the figure legend.**

E) Why glycostainning is labeling all proteins similarly to coomassie? Authors should provide a control with cells under PNGase, Endoglycosidase or any hydrolase that would work with your cells. Likewise, I don't understand the discrepancy between glycostanning and mass spec results (that cannot detect any glycopeptides from aglB- cells). Is that related only to O-glycosylation? Please clarify in the text.

> **Unfortunately, no endoglycosidases or hydrolases that are known to remove *Haloferax volcanii* glycans are known so far, leaving chemical deglycosylation as the only option, which we are currently not equipped to perform due to the toxic nature of this process. However, in addition to the non-glycosylated proteins of the CandyCane glycoprotein marker, a recombinantly expressed non-glycosylated protein has been included as a control. This allowed us to adjust exposure times so that signals from non-specific staining are reduced to a minimum, showing signals corresponding to glycoproteins more clearly.**
> **The perceived discrepancy between the glycostaining and results of the mass spectrometric analysis are now discussed more clearly. Glycoprotein staining of the Δ*aglB* strain is most likely due to either *O*-glycosylation or a third type of *N*-glycosylation, both of which have been reported previously.**

Lines 198-201: Please clarify how equal culture volumes yield a quantitative comparison between mid- and late-exponential growth phases. Higher the OD, higher the density, so different N of cells?

> **We have clarified the phrasing to explicitly state that quantitative analyses were not pursued and that the experimental setup was designed for a qualitative comparison between the analyzed strain.**

Lines 236-237: I don't understand how authors can infer that the vast majority of glycopeptides were identified. Was that calculated from the predicted glycoproteins? Please clarify.

> **We apologize for the confusion and rephrased the sentence to clarify that not the vast majority of all existing *N*-glycopeptides was meant, but the vast majority of identified *N*-glycopeptides**

Figure 3: Panels A-B relate to the total pool of peptides/proteins, while panels C-D zoom in into SLG. I think it would help the reader if panels C-D had SLG labeled directly in the figure.

Additionally, I have struggled to understand whether all AglB- and Agl15-dependent glycomodifications were detected in all SLG molecules, or there was a mixed population of different modified SLG molecules. If the latter follows, it would be incredibly exciting to be discussed here whether it points out to a spatiotemporal regulation within individual cells or different profiles during shape transitions throughout growth phases.

Panels C and D in Fig. 3 have been labeled to clarify that they depict the SLG. As stated in the results (lines 285-291), a population of peptides with either AglB- or Agl15-dependent glycosylation was found but, despite our best efforts, including analyzing results from an open modification search, no evidence for single molecules modified by both *N*-glycosylation pathways could be found.
We furthermore extended the discussion on the involvement of *N*-glycosylation in cell shape determination to include the potential for spatiotemporal regulation.

Figure 5: I recommend replace "reveals conservation within haloarchaea" with "reveals conservation restricted to haloarchaea". Why did the authors compare to Thaumaarchaeota and not other archaeal families? Please clarify in the text.

We have replaced "reveals conservation within haloarchaea" with "reveals conservation mostly restricted to haloarchaea".
We have clarified that all taxonomic phyla which are represented in OrthoDB have been included in the analysis. Phyla were only indicated when more than one taxonomic class exists within the phylum (which is the case for Euryarchaeota and Thaumarchaeota), but are now specified for all phyla for which at least one ortholog was found, i.e. for all phyla that are represented in the figure.

Line 582: I believe authors have a typo. Instead of 0.03, shouldn't be 0.3 for mid-exponential growth?

This has been corrected to 0.3

Strain List: There is little information about the strains used in the study and how they were made/confirmed.

Generation of the *N*-glycosylation pathway mutant strains by the Eichler laboratory has been described previously and we made sure that this origin is explicitly stated.
